# Light Absorption-Enhanced Ultra-Thin Perovskite Solar Cell Based on Cylindrical MAPbI_3_ Microstructure

**DOI:** 10.3390/ma17246284

**Published:** 2024-12-23

**Authors:** Wenfeng Fu, Chong Pan, Aixuan Zhou, Pengcheng Shi, Zao Yi, Qingdong Zeng

**Affiliations:** 1 School of Mathematics and Science, Joint Laboratory for Extreme Conditions Matter Properties, The State Key Laboratory of Environment-Friendly Energy Materials, Tianfu Institute of Research and Innovation, Southwest University of Science and Technology, Mianyang 621010, China; fuwenfeng2021@163.com (W.F.); 18990861683@163.com (A.Z.); pengchengshi114@163.com (P.S.); 2 State Grid Sichuan Electric Power Company, Chengdu Power Supply Company, Chengdu 610051, China; enoch19861212@126.com; 3 School of Physics and Electronic-Information Engineering, Hubei Engineering University, Xiaogan 432000, China; zengqingdong2005@163.com

**Keywords:** MAPbI_3_, cylinder, perovskite solar cells, performance optimization, solar energy absorption

## Abstract

In order to promote power conversion efficiency and reduce energy loss, we propose a perovskite solar cell based on cylindrical MAPbI3 microstructure composed of a MAPbI_3_ perovskite layer and a hole transport layer (HTL) composed of PEDOT:PSS. According to the charge transport theory, which effectually increases the contact area of the HTL, promoting the electronic transmission capability, the local field enhancement and scattering effects of the surface plasmon polaritons help to couple the incident light to the solar cell, which can increase the absorption of light in the active layer of the solar cell and improve its light absorption efficiency (LAE). based on simulation results, a cylindrical microstructure of the perovskite layer increases the contact area of the hole transport layer, which could improve light absorption, quantum efficiency (QE), short-circuit current density (J_SC_), and electric power compared with the perovskite layer of other structures. In the AM 1.5 solar spectrum, the average light absorption efficiency is 93.86%, the QE is 80.7%, the J_SC_ is 24.50 mA/cm^2^, and the power conversion efficiency (PCE) is 20.19%. By enhancing the efficiency and reducing material usage, this innovative design approach for perovskite solar cells is expected to play a significant role in advancing solar technology and positively impacting the development of renewable energy solutions.

## 1. Introduction

In the past ten years, perovskite materials have demonstrated excellent photoelectric properties due to their unique combination of high absorption coefficients (10^4^ cm^−1^) [1,2,3,4,5,6], long carrier diffusion lengths, and the ability to have their band gap tuned. These characteristics allow for optimization across different applications, such as photovoltaics, light emission, and heating. Common heterojunction perovskite solar cells (PSCs) have two structures of positive heterojunctions and an inverted heterojunction structure: an n-i-p structure and a p-i-n structure. Guo et al. first proposed inverted p-i-n-structured PSCs with a PCE of 3.9% [6,7]. In 2021, the highest PCE value of p-i-n PSCs was 20.6%, as studied by Jia Li et al. [8]. As of recent advancements, the highest reported PCE (power conversion efficiency) for p-i-n-structured perovskite solar cells (PSCs) has reached 26.41%, achieved by a research team at Tsinghua University in early 2024. This milestone was accomplished through the development of novel hole transport materials and the use of vacuum-deposited perovskite film [9]. Inverted p-i-n-structured perovskite cell devices can form films at lower temperatures, and have been used in high-efficiency silicon solar cells to make laminated solar cells to achieve light absorption at wider bands [10,11,12]. As one of the future development trends regarding composite flexible solar cells, inverted p-i-n PSCs are suitable for commercial production and application.

PEDOT:PSS is a polymeric material with high conductivity and adjustable electrical conductivity. PEDOT is a polymer of EDOT (3,4-ethylene dioxythiophene monomer), and PSS is a polystyrene sulfonate. Furthermore, PEDOT has good mechanical properties, allowing it to bend, stretch, curl, and tear. In inverted PSCs, PEDOT has a high ductility, high light transmittance, simple preparation, and can effectively transport holes to electrodes: PSS materials are often used in the HTL, increasing V_OC_ to a certain extent [13,14,15,16,17,18,19]. In 2024, Zhengguo Xiao, Cheng Bi et al. prepared PSCs using PEDOT:PSS with an optimal efficiency of 15.4% [6,20]. In 2019, Khan Mamun Reza et al. used solvent treatment PEDOT:PSS to prepare a MAPbI_3_ perovskite solar cell with a PCE of 18.18% [16]. PEDOT:PSS often requires dopants or additives to improve the efficiency of devices [18,21,22], which limits the improvement of photovoltaic performance of inverted PSCs. In order to increase the PCE of the cells, many scholars make nanostructures at the contact interface between the active layer and the HTL PEDOT:PSS, and improve the efficiency of the cell by increasing the contact area between the active layer and the HTL. Among the diverse range of nanostructures, nanocones, nanorods, nanorod holes, and nanorod columns have been documented in research studies [23,24]. In order to achieve the goal of reducing the overall thickness of the solar cell while enhancing its photoelectric conversion efficiency, we designed an ultra-thin inverted perovskite solar cell with tunable microstructures based on a MAPbI3 active layer [9]. This solar cell was developed on the basis of an inverted p-i-n-structured perovskite cell and features a cylindrical MAPbI3 structure. The cylindrical MAPbI3 structure combined with PEDOT:PSS increases the junction area, reduces the hole transmission distance, and reduces the composite probability of the electron–hole pair [25,26]. Compared to other nanostructures, nanorod columns offer a larger contact area, which reduces electron-hole pair recombination and decreases the hole transport distance. This facilitates the collection of holes by ITO, thereby enhancing the photovoltaic efficiency of the cell, but also shortens the hole transmission distance, which facilitates hole collection by ITO. In terms of optical loss, the nanometer cylinder can also increase the absorption of light inside the solar cell and reduce the overall thickness of the cell, but also improves the PCE.

## 2. Structure Design and Simulation

We use the optical wave characteristics in cylindrical MAPbI3 microstructure by using FDTD. To calculate the electromagnetic optical wave and observe the interaction with the perovskite solar cell structure, the Maxwell equation was used [27,28,29,30]. This method was chosen because of its broad spectrum of band, its computational power, and its high level of precision [31,32]. FDTD software (FDTD 8.15.736) based on Maxwell’s equation has been widely used to simulate solar cells [33,34,35]. Maxwell’s equation is expressed as:

(1) $$\frac{\partial E}{\partial t}=\frac{1}{\varepsilon }\nabla \times H-\frac{1}{\varepsilon }(J+\sigma E)$$

(2) $$\frac{\partial H}{\partial t}=-\frac{1}{\mu }\nabla \times E-\frac{1}{\mu }(M+\sigma_{m}M)$$

A reflectance monitor was placed at the top of the structure to calculate the reflectance coefficient, *R* [36,37,38]. Similarly, a transmission monitor was placed at the bottom of the structure to calculate the transmission coefficient *T*, so as to obtain the absorption rate of the entire solar cell [39,40,41]. The overall absorption rate can be calculated as:

*A* = 1 − *R* − *T* (3)

*T* is the transmission and *R* is the reflectivity, and the reflectivity is defined as the ratio of the intensity of the transmitted light. When the sun shines on the surface of the perovskite solar cell and makes vertical contact, its reflection coefficient can be expressed as [42,43]:

(4) $$R=\frac{{\left(n-1\right)}^{2}+k^{2}}{{\left(n+1\right)}^{2}+k^{2}}$$

n is the ratio of the speed of light in a vacuum to that in a semiconductor, and k is the extinction coefficient. Light scattering is a phenomenon that changes the transmission direction of light in the medium. In PSCs, light scattering can widen the light path and improve the exposure of the cell to sunlight, thus forming a secondary absorption.

FEM was conducted to determine the electrical parameters of PSCs, such as J_SC_, V_OC_, filling factor (FF), PCE, and Pmax [27,44,45,46]. To build the geometric model of the designed cell in FEM [47], define the modeling area material parameters, boundary conditions, scanning voltage and grid size, and finally run:

(5) $$V_{oc}=q^{-1}tK\ln\left(\frac{J_{sc}}{J}\right)$$

(6) $$J_{sc}=\frac{q}{2\pi \hbar c}\int_{280\;nm}^{800\;nm}\lambda Q_{E}(\lambda )I_{AM1.5}\lambda d_{\lambda }$$

(7) $$FF=\frac{P_{max}}{V_{oc}J_{sc}}$$

(8) $$PCE=\frac{P_{max}}{P_{in}}=\frac{V_{oc}\cdot J_{sc}\cdot FF}{P_{in}}$$

where Q is the charge of the electron, λ is the wavelength of the light source, C is the speed of light in a vacuum, Q_E_(λ) is the QE of the solar cells in the formula, and IAM1.5 represents the solar irradiance under AM1.5. The following formula can express the amount of photogenerated carriers per unit volume:

(9) $$G=\frac{Pabs}{2\Pi \hbar \omega }=-\frac{0.5Im\left\{\varepsilon \right\}}{\hbar }$$

Electron concentration is n and hole concentration is p. R means total carrier recombination. The simulation of electricity has four kinds of recombination mechanisms:

(10) $$R=R_{rad}+R_{Aug}+R_{SRH}+R_{surf}$$

(11) $$R_{rad}=B(n_{p}-n_{i}^{2})$$

(12) $$R_{Aug}=({nC}_{n}+{pC}_{p})(n_{p}-n_{i}^{2})$$

(13) $$R_{SRH}=\frac{np-n_{i}^{2}}{\tau_{p}(n+n_{t})+\tau_{n}(p+p_{t})}$$

(14) $$R_{surf}=\frac{np-n_{i}^{2}}{\frac{1}{S_{p}}(n+n_{ts})+\frac{1}{S_{n}}(p+p_{s})}$$

**Figure 1:**
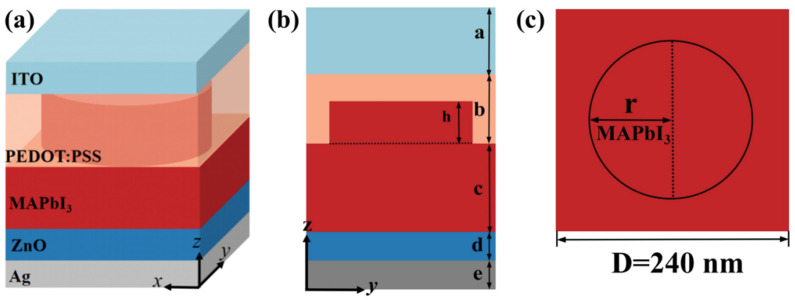
(**a**) The structure of the cylindrical MAPBI3 microstructure PSCs; (**b**) the side view of the designed structure; (**c**) the top view of the structure (period D = 240 nm).

In the FDTD simulation, Figure 1 illustrates a periodic structure with a period of D = 240 nm. The base consists of a 100 nm thick silver (Ag) layer, serving as the back electrode of the solar cell. Above this is a 100 nm thick ZnO layer, which functions as the electron transport layer (ETL) [48]. The main photoactive layer, made of CH_3_NH_3_PbI_3_(MAPBI_3_), is 500 nm thick and supports a cylindrical microstructure of the same material. This microstructure has a thickness h and a radius r. The hole transport layer (HTL) is composed of PEDOT:PSS, followed by a 250 nm thick ITO transparent conductive glass layer. A plane-wave light source ranging from 280 to 800 nm is used, with a mesh size of 5 nm.

**Figure 2:**
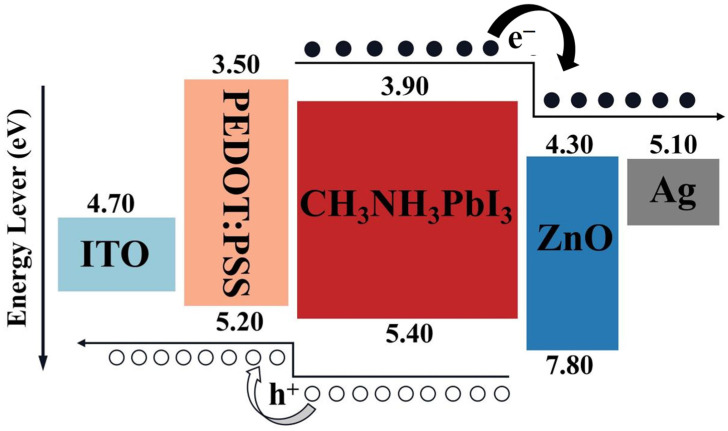
PEDOT:PSS/MAPBI_3_ organic–inorganic hybrid perovskite solar cell band map and schematic diagram of carrier transport under sunlight.

Figure 2 presents the energy band map and a schematic diagram of carrier transport in the designed perovskite solar cells (PSCs) with a MAPBI_3_ structure under sunlight. The organic hole transport layer (HTL) material, PEDOT:PSS, features an adjustable composition ratio, allowing for the tuning of the Fermi level position [49,50,51,52]. By adjusting the composition of various PEDOT:PSS solutions, a p-type PEDOT:PSS can be achieved for use as the HTL in our solar cell design [18,53,54,55]. The energy bands of MAPBI_3_ and PEDOT:PSS are closely aligned, resulting in a low transport barrier that facilitates easier electron and hole transport.

In conclusion, we performed optical simulation in FDTD to obtain the optical absorption curve, quantum efficiency curve, and integral current of the designed solar cell, using FEM technology in the electrical simulation [24,44,45], J_SC_, V_OC_, and PCE of the designed cell.

## 3. Results and Discussion

**Figure 3:**
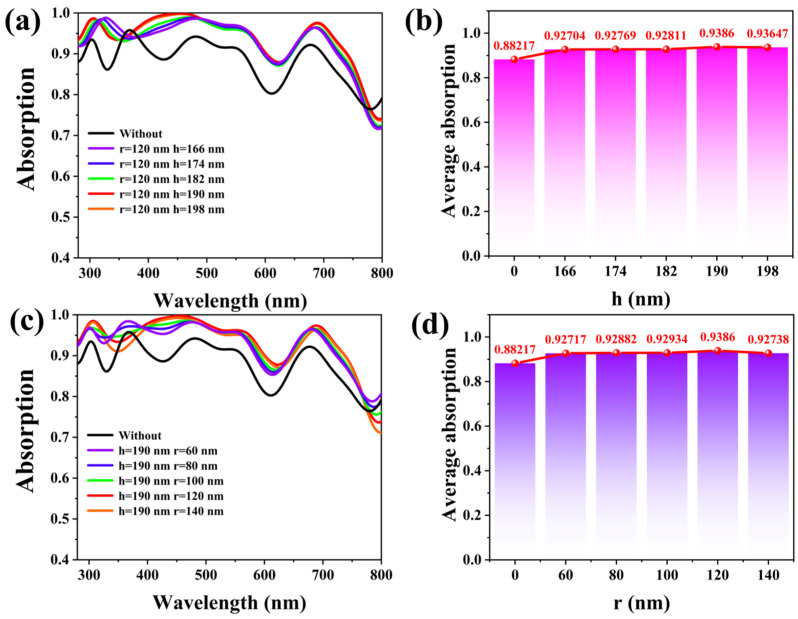
(**a**,**b**) Light absorption and average absorption with different heights of the cylindrical MAPBI3 structure (r = 120 nm; h = 166 nm,174 nm,182 nm,190 nm, and 198 nm); (**c**,**d**) light absorption and average absorption with different radiuses of the cylindrical MAPBI3 structure (r = 60 nm, 80 nm, 100 nm, 120 nm, and 140 nm).

In the FDTD, we investigated the effect of the cylindrical MAPbI3 geometry parameters on the optical properties of the designed hybrid PSCs. Figure 3a,b show the light absorption curves and the average absorption rate of the designed mixed PSCs under different heights of the cylindrical MAPbI3 structure. Due to the bandgap of CH3NH3PbI3 being 1.55 eV, the absorption curve shows a nearly identical trend within the wavelength range of 166 nm to 198 nm. But compared it with the average absorption rate shows, in the range of h, which is 166 to 198 nm, the trend in the average absorption of sunlight is increasing to decreasing. The maximum average absorption is 93.86% at h = 190 nm. Without the cylindrical MAPBI3 microstructure, the average absorption of PSCs is 88.22%, which is minimal. At the same time, we discuss the light absorption and the average adsorption rate of the designed cell with different cylindrical MAPBI3 structures. As Figure 3c shows, changing the cylindrical radius from 60 nm to 140 nm. At the 350 nm to 400 nm band, the light absorption of the cell decreases with the cylindrical radius increasing. From the 400 nm to 800 nm band, the light absorption of the designed cell increases with the increasing radius of the cylindrical MAPbI3 structure. Finally, Figure 3d shows that the average absorption increases with the cylindrical MAPBI3 structure radius increasing at r = 120 nm, which means that, when the nanocylinder of each cycle is a tangent, the average light absorption of the cell reaches its maximum.

**Figure 4:**
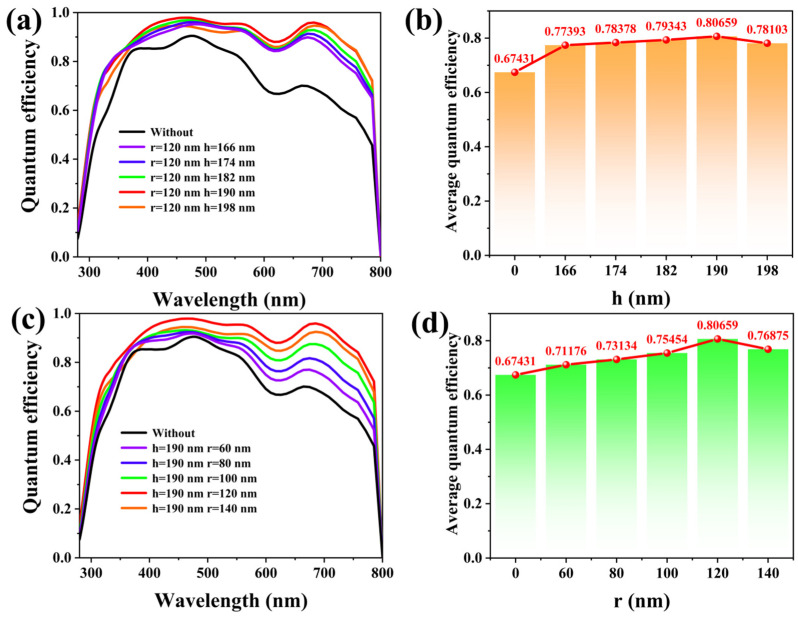
(**a**,**b**) QE and AQE with different heights of cylindrical MAPbI3 structure; (**c**,**d**) QE and AQE with different radiuses of cylindrical MAPbI3 structure.

Figure 4a,b show the quantum efficiency (QE) and average quantum efficiency (AQE), which measure the degree of the utilization of incident photons. It shows that the highest AQE is 80.66% and QE is best, on the whole, when h is 190 nm. Meanwhile, from 400 to 700 nm, the QE and AQE are relatively stable to use. In Figure 4c,d, we study the quantum efficiency and the average quantum efficiency of the designed cells at different cylindrical MAPbI3 structure radiuses. The cell exhibits the maximum QE when each cycle MAPbI_3_ cylinder is a tangent. When the radius r gradually increases to 120 nm, and the cycle MAPbI_3_ cylinder moves from phase to phase, the QE also gradually increases, but when r = 140 nm, when the MAPbI3 cylinder of each cycle intersects, the quantum efficiency of the solar cell decreases, and AQE is 76.87%. In conclusion, at h = 190 nm, r = 120 nm, the QE is the largest, which means the MAPbI_3_ layer not only enhances the light absorption and improves the rate of photogenerated carriers, but also increases the QE of the designed PSCs.

**Figure 5:**
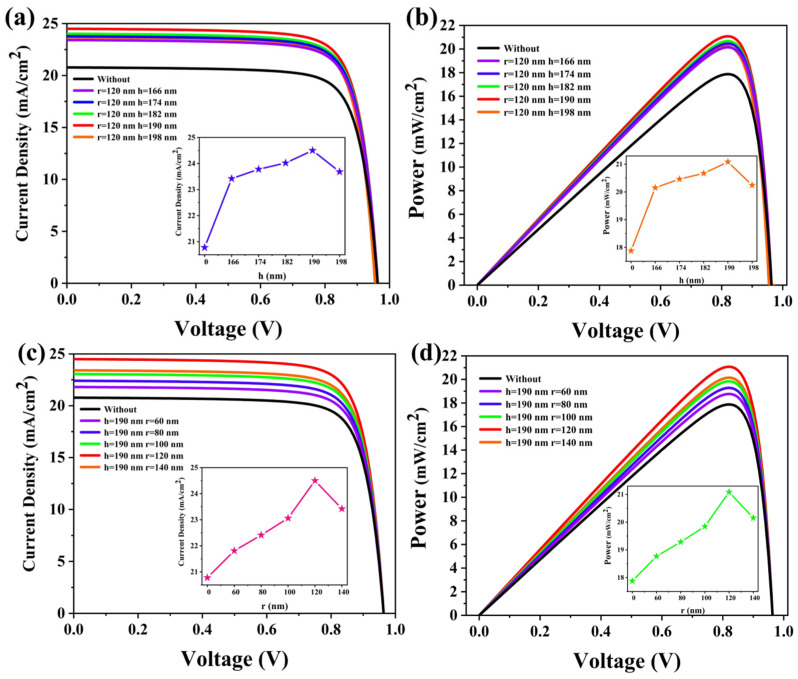
(**a**,**b**) The J_SC_ and electric power curves of the designed cells with different cylindrical MAPbI3 structure heights; (**c**,**d**) the short-circuit current density and electric power curves of the designed batteries with different cylindrical MAPbI3 structure radiuses.

We used the finite element method (FEM) to simulate the electrical characteristics of the device, incorporating various geometric parameters like radius and height, as selected in the FDTD optical simulation [56,57,58]. Figure 5a illustrates the relationship between the short-circuit current density (J_SC_) and the height of the cylindrical MAPbI3 structure, while Figure 5c depicts the relationship between J_SC_ and the radius of the MAPbI_3_ cylinders. The results indicate an improvement in J_SC_ with the cylindrical MAPbI_3_ structure. Figure 5b,d show the impact of these parameters on electrical power. The MAPbI_3_ cylinder reduces electron–hole pair recombination, enhancing performance. Specific parameters, such as cylinder height and associated J_SC_ and power conversion efficiency (PCE), are detailed in Table 1: for r = 60 nm, J_SC_ = 21.81 mA/cm^2^, and PCE = 20.15%. For r = 120 nm, J_SC_ = 24.50 mA/cm^2^, and PCE = 21.09%. Additional solar cell parameters are listed in Table 2. Overall, the inclusion of MAPbI_3_ cylinders significantly enhances the PCE of the designed cell and facilitates electron transport and collection.

**Figure 6:**
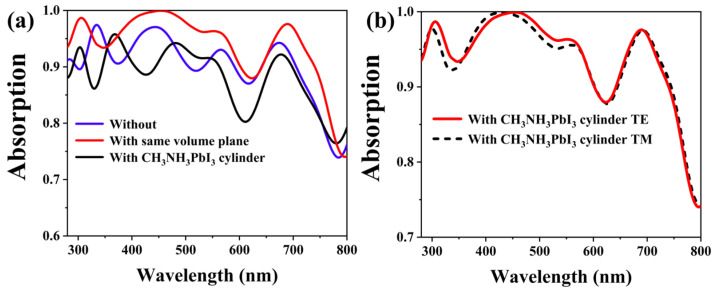
(**a**) The light absorption spectrum of PSCs with and without a cylindrical MAPbI3 structure and the planar MAPbI_3_ cells with the same volume; (**b**) the light absorption spectrum of a cylindrical MAPbI3 PSC in different polarization states of TE and TM.

In order to further verify the influence of the cylindrical MAPbI3 structure on the optical characteristics of such hybrid PSCs, we specially converted the MAPbI_3_ cylinder into a MAPbI3 plane of the same volume for comparative analysis. Figure 6a reveals the light absorption relationship among the three surfaces. In Figure 6b, the surfaces with cylindrical MAPbI3 PSCs have a broadband absorption of 300–800 nm in both polarization modes: TE and TM. The red curve is the light absorption of the cylindrical MAPbI3 PSCs, the blue curve represents the light absorption curve of the planar MAPbI_3_ cells with the same volume, and the black curve represents the light absorption curve of flat PSCs that do not contain a MAPbI_3_ cylindrical structure. By comparing the red and black curves, we found that the light absorption of the cylindrical MAPbI3 PSCs is significantly higher than the planar PSCs. The light absorption of the blue one is also lower than the red. The contrast between the blue and red curves shows that light absorption is not only dependent on the increase in MAPbI_3_ material, but because the cylindrical MAPbI3 structure increases the light absorption of the cell. The light absorption enhancement of the solar cell is caused by the existence of a cylindrical MAPbI3 structure, which helps the formation of the light trap effect inside the solar cell, which can form multiple reflections and absorb the incident light again [59,60,61]. We similarly verified the optical properties of solar cells with cylindrical MAPbI3 PSCs in two different polarization modes, TE and TM, which proved that the optical performance of the designed PSCs was reliable.

**Figure 7:**
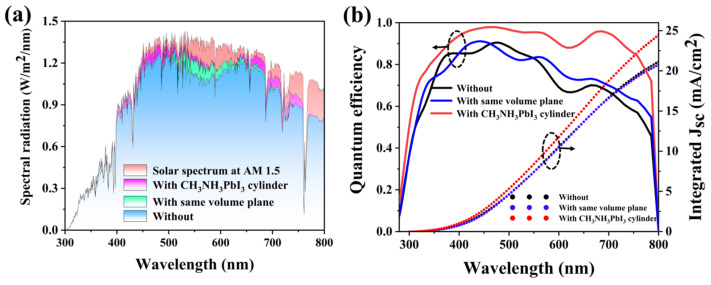
(**a**) The light absorption spectrum of the cell in the AM 1.5 solar spectrum with and without a MAPbI_3_ cylinder structure and with the cylinder replaced by a plane layer of the same volume; (**b**) the QE and integral current of the cell with and without a MAPbI_3_ cylinder structure and with the cylinder replaced by a plane layer of the same volume.

For further study of the response of hybrid PSCs of the planar type and the cylindrical MAPbI3 structure, with the same volume, we compared the absorption efficiency of these three in the AM 1.5 solar spectrum [62,63,64,65]. Figure 7a illustrates that PSCs featuring a cylindrical MAPbI3 structure exhibit the optimal response to sunlight. Through Figure 7b, we studied the relationship between quantum efficiency and integrated current among the three surfaces. Obviously, due to the embedding of the MAPbI_3_ structure, the quantum efficiency of the designed solar cell is greatly improved at 300–800 nm wavelengths, and the integral current is also enhanced, with an integral current of 24.48 mA/cm^2^. The J_SC_ of the planar perovskite solar cell is 20.78 mA/cm^2^. The integral current of the same volume plane layer is 21.16 mA/cm^2^. In conclusion, PSCs with a cylindrical MAPbI3 structure have the best optical performance.

**Figure 8:**
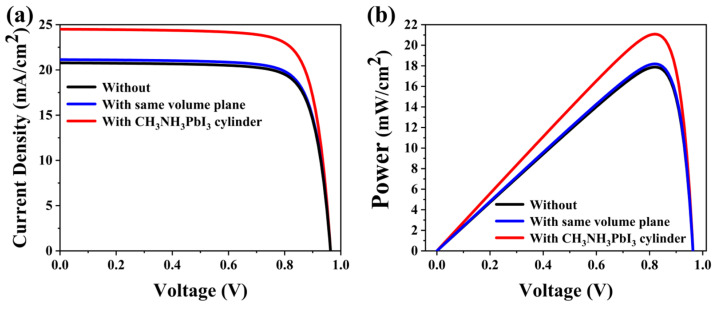
(**a**) The I-V curve of the solar cell with and without the cylindrical MAPbI_3_ structure and the cylinder under the optimal parameters of the cylindrical MAPbI_3_ structure; (**b**) The P-V curve of the solar cell with and without the cylindrical MAPbI_3_ structure and the cylinder with a plane layer of the same volume.

As shown in Figure 8a, the plane perovskite solar cell J_SC_ is 20.79 mA/cm^2^ and the perovskite solar cell J_SC_ is 21.22 mA/cm^2^. The J_SC_ of PSCs with a cylindrical MAPbI_3_ structure is 24.50 mA/cm^2^. For PSCs with the cylindrical MAPbI_3_ structure, the J_SC_ is greatly increased compared to those with a plane structure, from 20.79 mA/cm^2^ to 24.50 mA/cm^2^. As the plane PSCs J_SC_ is 21.22 mA/cm^2^, this indicates that the use of the same amount of MAPbI_3_ material will not greatly improve J_SC_, which proves the impact of the introduction of the cylindrical structure of MAPbI_3_ on the electrical performance of the solar cell [66,67,68]. As can be seen from Figure 8b, the Pmax of the flat perovskite solar cell is 17.88 mW/cm^2^. The Pmax is 18.16 mW with a plane layer of the same volume. The Pmax of the cell with a cylindrical MAPbI_3_ structure is 21.09 mW/cm^2^.

## 4. Conclusions

In the paper, we designed a hybrid perovskite solar cell with a cylindrical MAPbI_3_ microstructure on the basis of inverted p-i-n PSCs. The influence of the cylindrical MAPbI_3_ structure on the photoelectric performance of the designed organic–inorganic hybrid PSCs was explored by FDTD and FEM (finite element method). In the AM 1.5 solar spectrum, the average optical absorption rate of the hybrid PSCs with a cylindrical MAPbI_3_ structure is 93.86%. QE is 80.7%, which is higher than the average quantum efficiency of PSCs without cylinders. Also explored were the solar cells with a cylindrical MAPbI_3_ structure in TM and TE. In the electrical simulation, we calculated the important electrical parameters, the J_SC_ of hybrid PSCs with a cylindrical MAPbI_3_ structure is 24.50 mA/cm^2^, and the PCE is 20.19%; the J_SC_ of the plane cell with the same volume is 20.78 mA/cm^2^ and its PCE is 17.88%. PSCs with cylindrical MAPbI_3_ microstructures have high photoelectric performance, and the overall thickness of the cell is 1100 nm, which is ultra-thin and excellent performance. We have further reduced the thickness of the solar cells while improving its energy conversion efficiency, making it more competitive.

## Figures and Tables

**Table 1:** The specific parameters of the electrical performance of the designed batteries at different MAPbI_3_ cylinder heights.

h (nm)	V_OC_ (V)	J_SC_ (mA/cm^2^)	P (mW/cm^2^)
Planar	0.97	20.78	17.88
166	0.97	23.42	20.15
174	0.97	23.78	20.46
182	0.97	24.02	20.67
190	0.97	24.50	21.09
198	0.97	23.68	20.24

**Table 2:** The specific parameters of the electrical performance of the designed batteries under different cylindrical MAPbI3 radiuses.

r (nm)	V_OC_ (V)	J_SC_ (mA/cm^2^)	P (mW/cm^2^)
Planar	0.97	20.79	17.88
60	0.97	21.81	18.76
80	0.97	22.41	19.28
100	0.97	23.06	19.84
120	0.97	24.50	21.09
140	0.97	23.42	20.15

## Data Availability

The original contributions presented in this study are included in the article. Further inquiries can be directed to the corresponding author.
